# Digestible and Metabolizable Energy Intake in Humans: a Systematic Review

**DOI:** 10.1016/j.advnut.2026.100597

**Published:** 2026-02-06

**Authors:** Eiichi Yoshimura, Naoya Oi, Kanon Abe, Yuki Nishida

**Affiliations:** 1 Laboratory of Nutrition Metabolism, Research Center for Clinical Nutrition, National Institutes of Biomedical Innovation, Health and Nutrition, Settsu, Osaka, Japan; 2 Laboratory of Gut Microbiome for Health, Microbial Research Center for Health and Medicine, National Institutes of Biomedical Innovation, Health and Nutrition, Ibaraki, Osaka, Japan; 3 Department of Preventive Gerontology, Center for Gerontology and Social Science, National Center for Geriatrics and Gerontology, Obu, Aichi, Japan; 4 Department of Medicine and Science in Sports and Exercise, Graduate School of Medicine, Tohoku University, Sendai, Miyagi, Japan

**Keywords:** energy absorption, digestible energy intake, metabolizable energy intake, systematic review, bomb calorimeter

## Abstract

Understanding digestible energy intake (DEI) and metabolizable energy intake (MEI) is essential for elucidating human energy balance. The absolute of DEI refers to gross energy intake minus fecal energy loss (EL), whereas MEI further accounts for urinary EL. This systematic review aimed to synthesize the findings from studies that utilized bomb calorimetry to measure DEI and/or MEI (PROSPERO CRD42021230982). Medical Literature Analysis and Retrieval System Online (via PubMed), the Cochrane Library, Cumulative Index to Nursing and Allied Health Literature, and Scopus were searched for articles published between January 1973 and July 2024. Human studies (adults aged ≥18 y) were included without restrictions on study design. Data were descriptively summarized according to dietary conditions, including overeating, undereating, high-fiber diets, tree nut intake, time-restricted eating (TRE), medication use, and disease status. Twenty-three studies were included. Overeating generally increased absolute fecal ELs; however, proportional DEI and MEI remained relatively stable, suggesting adaptive responses. High-fiber diets and tree nut intake consistently lowered the proportions of DEI and MEI, indicating that dietary composition affects energy absorption efficiency. Results for TRE were inconsistent, with 1 study showing increased fecal EL and another reporting no significant changes. Aging and disease, particularly short bowel syndrome and home parenteral nutrition dependence, were associated with markedly reduced proportions of DEI and MEI. Despite methodological variability across studies, this review highlighted that both dietary quantity and composition significantly influence energy absorption. Furthermore, limited evidence suggested that aging and diseases impair energy absorption. Future studies using standardized protocols and randomized controlled trials are warranted to clarify the determinants of DEI and MEI across diverse populations.

This study was registered at PROSPERO as CRD42021230982.

**Statement of significance**

This study is the first to systematic review the absolute and relative values of digestible energy intake and metabolizable energy intake. The results of this study suggest that synthesize evidence on how dietary loads, contents, age, and disease status influence the absolute and proportional values of digestible energy intake and metabolizable energy intake, highlighting novel directions for future studies.

## Introduction

Despite global recognition of the health burden associated with excess body weight, no country has successfully reversed the increasing trends in adult overweight and obesity, which are projected to continue worldwide [1]. Concurrently, underweight and thinness remain serious public health challenges among school-aged children and adolescents in South Asia and parts of Africa [2]. In addition to these contrasting issues of overnutrition and undernutrition, unintentional weight loss due to disease contributes to the worsening of frailty in aging populations [3]. These examples highlight the importance of appropriate weight management across all age groups, socioeconomic settings, and disease management contexts. Therefore, maintaining a healthy body weight by avoiding both excess and insufficiency is a critical global challenge for improving well-being and extending healthy life expectancy.

Energy balance is a critical determinant of body weight regulation [4,5], and it involves the interplay between energy intake, energy losses (ELs) via feces and urine, and energy expenditure, including resting energy expenditure, thermic effects of food, physical activity, and disease or injury. According to the FAO of the United Nations [6], and the 2023 Dietary Reference Intakes for Energy [7], the absolute of digestible energy intake (DEI) refers to the gross energy ingested minus fecal EL. The absolute of metabolizable energy intake (MEI) is further subtracted from urinary EL, reflecting digestive and absorption capacity. Notably, energy produced by intestinal bacteria and gases in the gut must also be considered [6,7]. Although most research on energy balance has focused on energy intake and expenditure, the contribution of fecal and urinary energy excretion remains underexplored. Evaluating DEI and MEI provides valuable insights into nutrient absorption and the body’s effective utilization of energy.

Bomb calorimetry is one of the reference standard methods for evaluating DEI and MEI, enabling precise determination of the gross energy content of food, feces, and urine. It is particularly valuable for assessing the impact of dietary interventions on energy absorption rates, such as overeating and undereating, as well as specific eating patterns, such as time-restricted eating (TRE). Previous studies have highlighted that increased caloric intake does not directly translate to body weight gain, owing to adaptive increases in fecal ELs and thermogenic responses [8,9]. Conversely, during energy restriction, reduced ELs reflect a compensatory conservation mechanism. Recent evidence further suggests that the proportion of DEI or MEI may be higher during periods of overeating than during undereating [[10], [11], [12]], though the findings remain inconsistent. Moreover, the proportion of DEI and MEI appears to vary with age, disease status, diet type, and dietary composition. However, human studies using bomb calorimetry remain scarce because of the substantial burden on both participants and researchers, as complete collection of all food intake, feces, and urine >3–5 d is required.

The bomb calorimeter was first developed in the 1870s [13], and was later used by Atwater to evaluate the energy content of foods, leading to the well-known “Atwater system,” which assigns 4 kcal/g for carbohydrates and proteins, 9 kcal/g for lipids, and 7 kcal/g for alcohol [14,15]. Although the Atwater system remains a foundation in modern nutrition science, several limitations, including methodological constraints, such as issues related to the measurement conditions at the time, have been identified [16]. Consequently, energy conversion factors have been revised through subsequent experimental evaluations [6,17]. Despite these revisions, research on DEI and MEI remains fragmented. Given that life expectancy, dietary patterns, and disease structures have changed substantially since the time of Atwater, a comprehensive synthesis of existing studies is warranted to clarify current evidence gaps.

In this systematic review, we aimed to synthesize findings from studies that utilized bomb calorimetry to measure DEI and/or MEI under various dietary conditions. Moreover, we aimed to advance understanding of energy balance and inform evidence-based approaches for weight management and metabolic health by examining the effects of dietary interventions, specific nutrients, medications, and diseases on DEI and MEI.

## Methods

### Search strategy

Online databases, such as Medical Literature Analysis and Retrieval System Online (via PubMed), the Cochrane Library, Cumulative Index to Nursing and Allied Health Literature (CINAHL), and Scopus, were searched to identify studies related to DEI and MEI assessed using bomb calorimetry. The search strategy combined free-text keywords and, where applicable, medical subject headings (MeSH) terms. Specifically, we searched for articles that explicitly mentioned “bomb calorimet∗” and those using combustion-related terms (burn∗ and combust∗) combined with excretion-related terms (fec∗, faec∗, deject∗, flux, and urin∗). Additional terms included keywords related to EL (e.g., “energy loss” and “caloric loss”) combined with dietary intake terms (e.g., “diet intake” and “energy intake”); absorption-related terms (e.g., “absorpt∗” and “malabsorp∗”) combined with energy terms (e.g., “energy”); and the term “apparent metabolizable energy,” all of which were added to ensure broader coverage. Boolean operators (AND, OR) were applied consistently across all databases. MeSH terms (e.g., “burns,” “feces,” “urine,” and “absorption”) were employed in PubMed to enhance search precision. In databases without MeSH (e.g., Scopus) or those with different indexing systems (e.g., CINAHL), we conducted searches using only the free-text keywords described above. Full search strings for each database are provided in Supplementary Material 1. The metabolizable energy (ME) values of foods measured by bomb calorimetry have been comprehensively compiled in the USDA’s Energy Value of Foods report, published in 1973 [18]. Therefore, the literature search was limited to studies published in English between 1973 and July 2024. The reference lists of the included articles were manually searched to identify additional relevant studies from the above databases and Google Scholar. The key articles identified were used for further research via the Web of Science database-cited reference function. The protocol for this review was registered with PROSPERO (https://www.crd.york.ac.uk/PROSPERO/view/CRD42021230982).

### Selection criteria

This review included studies involving human participants without restrictions on study design. Eligible studies are required to report both energy intake and fecal and/or urinary EL measured using a bomb calorimeter. The exclusion criteria were as follows: nonoriginal research articles, animal experiments, and articles in languages other than English.

### Screening and data extraction

The study selection process followed the PRISMA 2020 guidelines. Database searches as well as backward and forward citation searches were performed by a single reviewer (YN). Records retrieved from all database searches were imported into EndNote software for reference management and duplicate removal. The deduplicated records were then exported to Rayyan QCRI [19], a web-based systematic review software, for title and abstract screening. Two independent reviewers (YN and KA), blinded to each other’s decisions, performed the initial title and abstract screening. Conflicts were resolved through discussion and consensus between the 2 reviewers (YN and KA). Subsequently, a second screening of full texts was conducted independently and blindly by 2 reviewers (EY and NO). A third reviewer (YN) was consulted if disagreements occurred. Study details, including author, year of publication, study design, study population, sample size, age, sex, BMI, fecal and/or urinary EL, and methodological details, were extracted by 1 reviewer (NO) and documented in summary tables. These tables were subsequently checked for accuracy and completeness by a second reviewer (EY).

### Quality assessment and data synthesis

Two reviewers (NO and EY or YN or KA) independently assessed the risk of bias of the included original articles. Quality assessment of included studies was conducted using the Joanna Briggs Institute (JBI) Critical Appraisal Tools appropriate for each study design. The JBI checklist for quasi-experimental studies was applied to 1-arm intervention studies; the checklist for randomized controlled trials (RCTs) was used for studies identified as RCTs or crossover trials; and the checklist for analytical gross-sectional studies was used for the cross-sectional studies [[20], [21], [22]]. Disagreements were resolved through discussion or consultation with a third reviewer (EY or YN or KA). In accordance with current JBI and Cochrane recommendations, we did not calculate a total score or provide a single overall assessment [23,24]. Instead, to ensure transparency regarding potential biases in each study, we reported domain-level assessments (e.g., randomization, allocation concealment, blinding, and outcome measurement).

The data were synthesized narratively. Outcomes were extracted from each study for gross energy intake (GEI, kcal/d), fecal and urinary EL (kcal/d), and DEI (%), which was calculated by subtracting fecal EL from GEI and dividing by GEI, and MEI (%). MEI was calculated by subtracting urinary EL from DEI and dividing by DEI. If GEI, urinary EL, and fecal EL were reported but the DEI or MEI values were not provided, the estimated mean DEI and MEI were calculated using the reported mean values of GEI, urinary EL, and fecal EL. To summarize the current evidence, a bubble plot was generated to visually illustrate the relationship between age and DEI and MEI, including only participants representing the control conditions in each study.

## Results

### Description of studies

**FIGURE 1:**
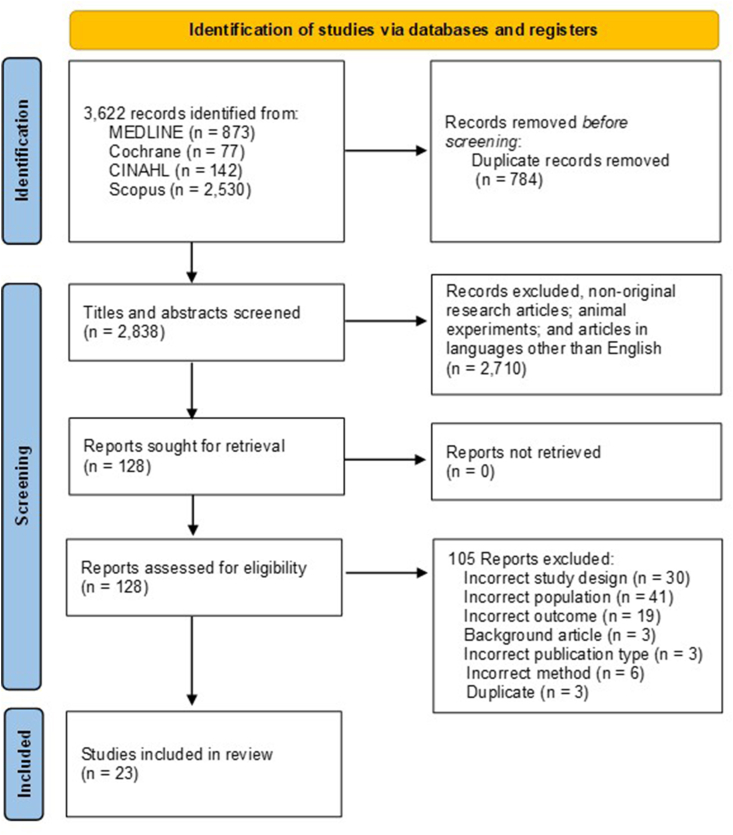
Flow diagram outlining the method used to determine studies for inclusion in this systematic review on energy absorption assessed via bomb calorimetry. CINAHL, Cumulative Index to Nursing and Allied Health Literature; MEDLINE, Medical Literature Analysis and Retrieval System Online.

The initial search yielded 3622 records, including 784 duplicates. Figure 1 is created in accordance with the PRISMA 2020 guidelines. We organized the exclusion reasons in the following order and revised them to match the number of adopted final articles: *1*) incorrect study design, *2*) incorrect population, *3*) incorrect outcome, *4*) background article, *5*) incorrect publication, and *6*) article in language other than English. Two authors (YN and KA) screened 2838 titles and abstracts, and 2710 articles were excluded following the exclusion criteria. Subsequently, 2 other authors (EY and NO) screened 128 full-text articles, and 105 articles were excluded for the following reasons: incorrect study design (*n* = 30), population (*n* = 41), study outcome (*n* = 19), background article (*n* = 3), incorrect publication type (*n* = 3), incorrect method (*n* = 6), and duplicate (*n* = 3). Overall, 23 articles were included in the final analyses [[8], [9], [10], [11], [12],[25], [26], [27], [28], [29], [30], [31], [32], [33], [34], [35], [36], [37], [38], [39], [40], [41], [42]].

### Quality appraisal

Seventeen studies were assessed using the JBI checklist for RCT, 2 studies using the analytical cross-sectional tool, and 4 studies using the quasi-experimental tool. In the study by Basolo et al. [11], a single paper reported both a separate RCT and a crossover study, which were evaluated separately. Regarding the RCTs, many studies did not clearly describe the methods of randomization (*n* = 12), allocation concealment (*n* = 15), or whether outcome assessors were blinded (*n* = 17). Additionally, in the 14 studies investigating the dose-response relationship between diet and DEI or MEI, blinding of both participants and investigators was not feasible due to the study design. In the cross-sectional studies, bias was noted mainly due to inadequate handling of confounders. The quasi-experimental studies were all published in the 1980s, and none reported information regarding the reliability of outcomes or measurement methods before and after the intervention. Detailed results of the risk of bias assessments are provided in Supplementary Tables 1–3.

### Study characteristics

Table 1 summarizes the characteristics of these studies. Of the 23 studies, 16 were conducted in the United States [8,10,11,[25], [26], [27],[29], [30], [31],^33^,[37], [38], [39], [40], [41], [42]], 5 in Europe [9,28,[34], [35], [36]], and 2 in Asia [12,32]. A total of 16 studies employed a crossover design [[10], [11], [12],[28], [29], [30], [31], [32], [33], [34],[36], [37], [38], [39],^42^], 4 studies were RCTs (1 was a combination of a crossover and RCT) [11,25,29,40,41], 4 studies were non-RCTs [8,9,25,27], 1 study was observational [26], and 1 study was cross-sectional study [35]. A total of 20 studies involved healthy adults or those without specific disease description [[8], [9], [10], [11], [12],[25], [26], [27], [28], [29], [30], [31], [32], [33],[37], [38], [39], [40], [41], [42]], whereas 2 focused on patients with short bowel syndrome (SBS) [34,36], and 1 reported on patients with intestinal failure [35]. Among intervention studies, 6 investigated dietary load (overeating and/or undereating), 6 on dietary content (high-fiber, high-protein/carbohydrate/fat diet, and low-glycemic diet), 4 on tree nuts (pistachios, walnuts, cashew nuts, and almonds), 3 on the effects of medication or supplementation (vancomycin, cholylsarcosine, and colostrum), 2 on time-restricted diet, and 1 on resistance training. Among the 529 participants across all studies, 295 were male (55.8%), 217 were female (41.0%), and 17 had unreported sex (3.2%) [8]. The mean age of the participants ranged from 21 to 68 y, with only 2 studies focusing on participants aged ≥60 y [34,40]. The mean BMI of participants ranged from 20.4 to 40.4 kg/m^2^, with 3 studies not reporting BMI data [8,26,28].

**TABLE 1:** Characteristics of the studies

Authors	Subject information	Study design	Study summary with a focus on DEI or MEI	Condition	*n*	M/F	Age	BMI
Webb, et al. (1983) [25] Hum Nutr Clin Nutr, United States	No description of disease	Nonrandomized controlled trial	Effect of overeating with different diet compositions on weight gain	High-protein and fat diet control	4	2/2	46.3 ± 6.3	25.5 ± 5.3
				High-protein and fat diet overeating				
				Mean diet control	4	2/2	48.3 ± 7.9	26.1 ± 7.7
				Mean diet overeating				
				High-carbohydrate diet control	4	2/2	44.5 ± 2.6	22.8 ± 5.3
				High-carbohydrate diet overeating				
Dallosso, et al. (1984) [9] Br J Nutr, United Kingdom	Healthy participants	1-arm intervention	Effects of overeating on 24-h energy expenditure, diet-induced thermogenesis, and DEI	Control	8	8/0	22.9 ± 2	21.9 ± 1.3
				Overeating				
Miles, et al. (1984) [26] Am J Clin Nutr, United States	Healthy participants	Observational study	Season changes in available energy for self-selected meals	Men	29	13/0	35.3	—
				Spring				
				Summer				
				Fall				
				Winter				
				All seasons				
				Women		0/16	34.2	—
				Spring				
				Summer				
				Fall				
				Winter				
				All seasons				
Webb, P., et al. (1985) [8] Int J Obes, United States	Free of disease	1-arm intervention	Effects of different dietary intake on food digestion and absorption	Control	17	—	40.5	—
				Overeating				
				Control				
				Undereating				
Miles, et al. (1986) [27] Hum Nutr Appl Nutr, United States	Healthy participants	1-arm intervention	Effect of underfeeding on metabolizable energy intake	All	—	—	—	—
				Control	9	5/4	34.9 ± 11.4	27.8 ± 3.8
				Mid-undereating				
				End-undereating				
				Male	—	—	—	—
				Control	5	5/0	35.2 ± 11.7	28.1 ± 4.7
				Mid-undereating				
				End-undereating				
				Female	—	—	—	—
				Control	4	0/4	34.5 ± 12.7	27.5 ± 3.2
				Mid-undereating				
				End-undereating				
Wisker, et al. (1988) [28] J Nutr, Germany	Healthy participants	Crossover trial	Effect of the intake of fiber between low- and high-fiber diets on metabolizable energy intake	Low-fiber diet	6	0/6	23∼27	—
				High-fiber diet				
Baer, et al. (1997) [29] J Nutr, United States	Healthy participants	Crossover trial	Effect of typical European and American mixed diets with different fat and fiber contents on metabolizable energy intake	High fat, high fiber	5	4/1	34.8 ± 11.7	26.2 ± 4.7
				High fat, medium fiber				
				High fat, low fiber				
				Medium fat, high fiber	6	2/4	31.2 ± 6.4	27.1 ± 8.6
				Medium fat, medium fiber				
				Medium fat, low fiber				
				Low fat, high fiber	6	3/3	33.3 ± 6.2	23.6 ± 3.7
				Low fat, medium fiber				
				Low fat, low fiber				
Heydorn, et al. (1999) [34] Scand J Gastroenterol, Denmark	Nonhealthy participants (short bowel syndrome)	Crossover trial	Effect of medication on MEI. Assessment for the utility of conjugated bile acid replacement with cholylsarcosine	Cholylsarcosine (0 g/d)	4	2/2	63.3 ± 10.7	22 ± 2.3
				Cholylsarcosine (6 g/d)				
				Cholylsarcosine (12 g/d)				
Jeppesen, et al. (2000) [35] Gut, Denmark	Nonhealthy participants (45 HPN patients with intestinal failure and in 44 non-HPN borderline patients with a short bowel or malabsorption exceeding 2 MJ/d)	Cross-sectional study	Impact of home parenteral nutrition patients with intestinal failure on MEI	Non-HPN	44	24/20	48	22.1
				HPN	45	31/14	49	20.8
Campbell, et al. (2002) [40] Metabolism, United States	Healthy participants	Randomized controlled trial	Effect of resistance training on the MEI required by healthy, free-living, older people to achieve and to maintain stable body weights during a 14-wk period of precise dietary control	Sedentary (SED)	—	—	—	—
				Baseline	10	4/6	66±3	24.3
				Week RT6				
				Week RT12				
				Lower body resistance training (LBRT)	—	—	—	—
				Baseline	9	4/5	67±3	25.1
				Week RT6				
				Week RT12				
				Whole-body resistance training (WBRT)	—	—	—	—
				Baseline	9	3/6	67±1	26.8
				Week RT6				
				Week RT12				
				Men	—	—	—	—
				Baseline	11	11/0	68±2	25.8
				Week RT6				
				Week RT12				
				Women	—	—	—	—
				Baseline	17	0/17	66±2	25.1
				Week RT6				
				Week RT12				
Clapp, et al. (2007) [30] Metab Syndr Relat Disord, United States	Healthy participants	Crossover trial	Effect of different glycemic index carbohydrates on MEI	Low-glycemic index	7	0/7	35±8	26.6±2.9
				High-glycemic index				
Zou et al. (2007) [41] Am J Clin Nutr, United States	Healthy participants	Randomized controlled trial	Effect of Atwater factors on estimating MEI from low-fat and high-fiber diets under reduced intake	Refined diet	9	5/4	35.9 ± 2.1	25.8
				Fruit and vegetable diet	9	5/4	35.2 ± 3.7	26.8
				Cereal diet	9	5/4	38.8 ± 3.9	24.8
Jumpertz, et al. (2011) [10] Am J Clin Nutr, United States	Healthy participants	Crossover trial	Effects of varying nutrient load on MEI in lean and obese individuals	Lean	—	—	—	—
				2400 kcal/d	12	12/0	32.8±9.2	23.4±1.7
				3400 kcal/d				
				Obese	—	—	—	—
				2400 kcal/d	9	9/0	35.8±10.6	40.4±4.6
				3400 kcal/d				
Baer, et al. (2012) [31] Br J Nutr, United States	Healthy participants	Crossover trial	Effect of a balanced diet with pistachio on MEI	Control	18	9/9	50	27.9
				42 g/d of pistachios				
				84 g/d of pistachios				
Lund P, et al. (2012) [36] Eur J Clin Nutr, Denmark	Nonhealthy participants (short bowel syndrome)	Crossover trial	Effect of colostrum use on MEI in patients with short bowel syndrome	Baseline 1 (water)	12	7/5	55.7 ± 10.7	20.4 ± 3.1
				Treatment 1 (colostrum)				
				Baseline 2 (water)				
				Treatment 2 (control)				
Novotny, et al. (2012) [38] Am J Clin Nutr, United States	Healthy participants	Crossover trial	Effect of a balanced diet with almonds on MEI	Control	18	10/8	56.0±8.6	27.4±4.2
				Almonds (42 g/d)				
				Almonds (84 g/d)				
Baer et al. (2014) [42] J Nutr, United States	Healthy participants	Crossover trial	Effect of resistant maltodextrin on MEI and net energy using indirect calorimetry	placebo (0 g/d RM + 50 g/d maltodextrin)	15	15/0	47 ± 2	26.9 ± 0.8
				RM (25 g/d RM + 25 g/d maltodextrin)				
				RM (50 g/d RM + 0 g/d maltodextrin)				
Baer, et al. (2016) [37] J Nutr, United States	Healthy participants	Crossover trial	Effect of a balanced diet with walnuts on MEI	Base diet	18	10/8	53.1±2.2	28.8±0.9
				Walnuts				
Baer, et al. (2018) [39] Nutrients, United States	Healthy participants	Crossover trial	Effect of a balanced diet with cashew nuts on MEI	Control diet	18	9/9	56.9 ± 2.4	28.4 ± 1.1
				Control diet + cashew diet				
Basolo A, et al. (2020) [11] Nat Med, United States	Healthy participants	Crossover trial	Effect of different dietary loads and antibiotics on MEI	ALL	—	—	—	—
				Underfeeding	27	17/10	35.1±7.3	32.3±8.0
				Overfeeding				
				Male	—	—	—	—
				Underfeeding	17	17/0	36.0 ± 7.1	32.2 ± 8.1
				Overfeeding				
				Female	—	—	—	—
				Underfeeding	10	0/10	33.6 ± 7.8	32.5 ± 8.1
				Overfeeding				
				ALL	—	—	—	—
				Vancomycin group	13	8/5	33.5 ± 8.3	33.5 ± 8.4
				Placebo	14	9/5	36.6 ± 6.2	31.2 ± 7.7
Bao, et al. (2022) [32] Front Endocrinol, China	Healthy participants	Crossover trial	Effect of TRE (5.5 h meal period) and control (11 h meal period) schedules on MEI	Control	12	5/7	24±2.3	21.9 ± 1.71
				TRE				
Dawson, et al. (2024) [33] Cell Rep Med, United States	Healthy participants	Crossover trial	Effect of early TRE (6-h meal period) and control (12-h meal period) schedules on MEI	Early TRE	16	8/8	31.1±5.2	23.8 ± 3.4
				Control				
Yoshimura, et al. (2024) [12] Obesity (Silver Spring), Japan	Healthy participants	Crossover trial	Effects of different energy loads on MEI	Overfeeding	10	10/0	21.0 ± 1.0	20.8 ± 2.3
				Control				
				Underfeeding				

Abbreviations: DEI, digestible energy intake; HPN, home parenteral nutrition; MEI, metabolizable energy intake; TRE, time-restricted eating; RM, resistant maltodextrin; RT6 (6 wk from intervention study); RT12 (12 wk from intervention study).

The effects of energy loads, dietary contents, TRE, and clinical conditions on the proportion of DEI and MEI are summarized qualitatively (Table 2). To ensure consistency across heterogeneous study designs, we report overall trends rather than individual mean ± SD or *P* values.

**TABLE 2:** DEI and MEI and summary of results

Authors	Condition	Energy loss via urine (kcal/d)	Energy loss via feces (kcal/d)	Energy loss via feces and urine (kcal/d)	DEI (%)	MEI (%)	Summary
Webb, et al. (1983) [25] Hum Nutr Clin Nutr, United States	High-protein and fat diet control	125 ± 31	171 ± 42	296 ± 37	92.7 ± 3.1	87.7 ± 3.2	Values are expressed as mean (±SD). The absolute of energy loss in urine and feces generally increased during overeating, but in percentage terms there was less undigested food during overeating than during control (not the result of a statistical analysis between condition).
	High-protein and fat diet overeating	121 ± 31	192 ± 104	313 ± 125	94.6 ± 2.4	91.1 ± 2.8	
	Mean diet control	74 ± 35	201 ± 43	274 ± 77	91.3 ± 0.7	88.2 ± 1.2	
	Mean diet overeating	97 ± 44	193 ± 87	290 ± 130	94.3 ± 1.8	91.5 ± 2.7	
	High-carbohydrate diet control	85 ± 12	217 ± 136	301 ± 142	92.4 ± 2.7	89.2 ± 2.3	
	High-carbohydrate diet overeating	109 ± 21	302 ± 71	411 ± 88	91.8 ± 1.4	88.8 ± 1.4	
Dallosso, et al. (1984) [9] Br J Nutr, United Kingdom	Control	104 ± 21	165 ± 27	269 ± 37	94.8 ± 0.8	91.6 ± 0.8	Values are expressed as mean (±SD). With overfeeding, there was a significant increase in daily feces energy excretion. However, when expressed as a proportion of the gross energy intake, the proportion of DEI was similar on control and overfeeding (95.6% vs. 94.8%, not shown in *P* value).
	Overeating	87 ± 18	209 ± 61	296 ± 71	95.6 ± 1.2	93.8 ± 1.4	
Miles, et al. (1984) [26] Am J Clin Nutr, United States	Men	—	—	—	—	—	Values are expressed as mean (±SD). The proportion of DEI and MEI was calculated from the mean of intake and excretion from urine and feces. The digestibility of these diets ranged from 87% to 98% (mean±SD, 93.7%±2.3%). Statistical analysis results between seasons were not shown.
	Spring	93±26	136±44	229	94.0	89.9	
	Summer	97±21	139±34	236	94.1	90.0	
	Fall	106±22	145±49	251	93.8	89.2	
	Winter	108±20	152±43	260	93.5	88.9	
	All seasons	101±23	143±42	244	93.9	89.5	
	Women	—	—	—	—	—	
	Spring	70±13	97±34	167	94.3	90.2	
	Summer	73±23	90±30	163	94.7	90.5	
	Fall	76±13	103±41	179	93.9	89.4	
	Winter	76±14	110±43	186	93.3	88.7	
	All seasons	74±16	110±37	184	93.5	89.1	
Webb, P., et al. (1985) [8] Int J Obes, United States	Control	94	196	290	92.1	88.4	Values are expressed as mean. The proportion of DEI and MEI was calculated from the mean of intake and excretion from urine and/or feces. There was no difference in the digestibility of the diets between the 2 types of participants, and their thermogenic response appeared to be identical. The proportion of DEI and MEI was higher in overeating than control and undereating (not the result of a statistical analysis).
	Overeating	109	229	338	93.5	90.0	
	Control	153	249	402	91.3	86.0	
	Undereating	121	181	302	90.0	84.0	
Miles, et al. (1986) [27] Hum Nutr Appl Nutr, United States	All	—	—	—	—	—	Values are expressed as mean (±SD). The absolute/proportion of DEI and MEI was calculated using the individual subject’s intake and excretion from urine and feces. No significant difference in the 3 energy nutrient coefficients of availability between males and females was observed.
	Control	149 ± 33	249 ± 95	398 ± 106	91.3 ± 2.9	86.1 ± 2.9	
	Mid-undereating	125 ± 38	198 ± 47	323 ± 45	88.7 ± 3.9	82.0 ± 4.0	
	End-undereating	117 ± 44	163 ± 37	280 ± 63	91 ± 2.5	84.7 ± 3.3	
	Male	—	—	—	—	—	
	Control	163 ± 39	289 ± 109	452 ± 111	90.7 ± 3.6	85.5 ± 3.4	
	Mid-undereating	144 ± 43	202 ± 51	346 ± 23	90.2 ± 3.2	83.6 ± 2.5	
	End-undereating	132 ± 55	178 ± 31	310 ± 59	91.6 ± 1.6	85.6 ± 1.3	
	Female	—	—	—	—	—	
	Control	131 ± 10	199 ± 46	330 ± 50	92.1 ± 2.1	86.9 ± 2.4	
	Mid-undereating	100 ± 8	193 ± 50	293 ± 50	86.8 ± 4.2	80.1 ± 5.1	
	End-undereating	98 ± 12	144 ± 38	242 ± 49	90.2 ± 3.5	83.6 ± 4.9	
Wisker, et al. (1988) [28] J Nutr, Germany	Low-fiber diet	109 ± 13	144 ± 42	253 ± 45	93.2	88.0	Values are expressed as mean (±SD). The proportion of MEI was calculated from the mean of intake and excretion from urine and feces. Compared with the low-fiber diet, the absolute of feces energy loss increased during consumption of the high-fiber diet (*P* < 0.001). The proportion of DEI was lower in high-fiber diet than low-fiber diet (86.9 % vs. 93.2 %, statistical analysis results between conditions were not shown).
	High-fiber diet	102 ± 17	307 ± 101	409 ± 100	86.9	82.5	
Baer, et al. (1997) [29] J Nutr, United States	High fat, high fiber	125 ± 5	265 ± 17	390	90.9	88.4 ± 0.6	Values are means ± pooled SEMs. Gross energy intake was estimated from the absolute of MEI and energy excretion (feces and urine). The proportion of DEI and MEI was calculated from their representative values. The proportion of MEI was lower when subjects consumed the high-fiber diets compared with the low-fiber diets (*P* < 0.05).
	High fat, medium fiber	122 ± 5	210 ± 17	332	92	89.0 ± 0.6	
	High fat, low fiber	122 ± 5	115 ± 17	237	95.8	92.2 ± 0.6	
	Medium fat, high fiber	111 ± 5	171 ± 17	282	92.8	89.6 ± 0.6	
	Medium fat, medium fiber	102 ± 5	129 ± 17	231	94.6	91.2 ± 0.6	
	Medium fat, low fiber	126 ± 5	96 ± 17	222	96.1	91.6 ± 0.6	
	Low fat, high fiber	139 ± 5	231 ± 17	370	89.9	86.3 ± 0.6	
	Low fat, medium fiber	143 ± 5	133 ± 17	276	94.6	90.0 ± 0.6	
	Low fat, low fiber	146 ± 5	128 ± 17	274	94.7	89.9 ± 0.6	
Heydorn, et al. (1999) [34] Scand J Gastroenterol, Denmark	Cholylsarcosine (0 g/d)	—	1481	—	66	—	Values are expressed as mean. There were no statistically significant differences in the proportion of DEI among the 3 groups.
	Cholylsarcosine (6 g/d)	—	1385	—	69		
	Cholylsarcosine (12 g/d)	—	1433	—	68		
Jeppesen, et al. (2000) [35] Gut, Denmark	Non-HPN	—	764 (540, 1130)	—	71 (58, 78)	—	Values are expressed as median (25–75 percentiles). When enteral nutrition was added, the proportion of DEI was 71% in non-HPN patients and 49% in HPN patients (*P* < 0.01).
	HPN		993 (306, 1340)		49 (40, 76)		
Campbell, et al. (2002) [40] Metabolism, United States	Sedentary (SED)	—	—	—	—	—	Values are expressed as mean (±SD). The absolute/proportion of DEI and MEI was calculated from the mean of intake and excretion from urine and/or feces. The dietary gross energy intake and the absolute of MEI were increased over time to achieve and maintain constant body weight among the subjects. These increases over time were necessary in all 3 groups (i.e., there were no significant group-by-time interactions). The absolute of MEI in the SED, LBRT, and WBRT groups was increased by 17%±5%, 14%±7%, and 12%±7%, respectively. Gross energy excretions in urine and feces did not change over time in any of the groups. Statistical analysis results regarding the proportion of MEI between conditions were not shown.
	Baseline	62 ± 7	535 ± 36	597	79.0	76.5	
	Week RT6	67 ± 5	590 ± 38	657	78.8	76.4	
	Week RT12	62 ± 7	523 ± 72	585	81.6	79.4	
	LBRT	—	—	—	—	—	
	Baseline	74 ± 12	571 ± 88	645	78.0	75.2	
	Week RT6	69 ± 7	654 ± 84	714	76.2	74.1	
	Week RT12	72 ± 7	580 ± 76	652	80.0	77.5	
	WBRT	—	—	—	—	—	
	Baseline	60 ± 5	559 ± 36	619	77.6	75.2	
	Week RT6	76 ± 7	616 ± 48	692	77.4	74.6	
	Week RT12	72 ± 5	544 ± 41	616	79.7	77.0	
	Men	—	—	—	—	—	
	Baseline	79 ± 7	669 ± 57	748	76.8	74.1	
	Week RT6	76 ± 5	685 ± 60	761	78.9	76.5	
	Week RT12	79 ± 5	635 ± 67	714	81.3	79.0	
	Women	—	—	—	—	—	
	Baseline	57 ± 5	480 ± 24	537	79.3	76.8	
	Week RT6	67 ± 5	576 ± 36	643	76.4	73.7	
	Week RT12	60 ± 5	490 ± 36	550	79.8	77.3	
Clapp, et al. (2007) [30] Metab Syndr Relat Disord, United States	Low-glycemic index	—		313±72	—	87.4	Values are expressed as mean (±SD). The proportion of MEI was calculated from the mean of intake and excretion from urine and feces. The absolute of MEI was significantly higher on the low-glycemic diet than the high-glycemic diet (*P* < 0.05). Statistical analysis results regarding the proportion of DEI between conditions were not shown.
	High-glycemic index			151±21		93.3	
Zou et al. (2007) [41] Am J Clin Nutr, United States	Refined diet	93 ± 5	191 ± 14	284	93.5 ± 0.19	90.3	Values are means ± pooled SEMs. The proportion of MEI was calculated from the mean of the results. The proportion of DEI was lower with the higher-fiber diets (the fruit and vegetable diet and the cereal diet) than the low-fiber diets (the refined diet) (*P* < 0.001).
	Fruit and vegetable diet	94 ± 7	267 ± 38	361	90.0 ± 0.79	86.2	
	Cereal diet	100 ± 10	255 ± 29	355	89.1 ± 0.23	84.8	
Jumpertz et al. (2011) [10] Am J Clin Nutr, United States	Lean	—	—	—	—	—	Values are expressed as mean (±SD). The absolute/proportion of MEI was calculated from the mean of intake and excretion from urine and feces. The individual difference in the proportion of DEI with the 3400 kcal/d diet compared with that with the 2400-kcal/d diet was significant in the case of lean individuals but not for individuals with obesity [+1.3%±1.9% (*P* = 0.04) and +0.2% ±1.2% (*P* = 0.59), respectively].
	2400 kcal/d	88.4±30.8	134.3±48.9	222.7	95.1±1.8	91.9	
	3400 kcal/d	106. 0±34.1	145.1±42.7	251.1	96.2±1.1	93.6	
	Obese	—	—	—	—	—	
	2400 kcal/d	99.3±31. 2	133.2±44.8	232.5	95.2±1.4	91.7	
	3400 kcal/d	112.6±28.4	173. 7±65.0	286.3	95.4±1.8	92.5	
Baer, et al. (2012) [31] Br J Nutr, United States	Control	139	131±13.3	270	94.9	89.5 ± 0.4	Values are means ± pooled SEMs. DEI was calculated from the mean of the results. The absolute of feces energy loss significantly increased with the addition of pistachios to the diet. The proportion of MEI significantly decreased with both of pistachios than control. The dose size of pistachios did not affect the proportion of MEI, as these values were similar between the 42 and 84 g/d of pistachios.
	42 g/d of pistachios	150	181±13.3	331	93.1	87.4 ± 0.4	
	84 g/d of pistachios	133	221±13.3	354	91.8	86.8 ± 0.4	
Lund P, et al. (2012) [36] Eur J Clin Nutr, Denmark	Baseline 1 (water)	—	1433 ± 812	—	56 ± 21	—	Values are expressed as mean (±SD). No difference in the absolute or proportion of DEI was observed between colostrum and control treatments.
	Treatment 1 (colostrum)	—	1650 ± 862	—	57 ± 19	—	
	Baseline 2 (water)	—	1299 ± 769	—	60 ± 22	—	
	Treatment 2 (control)	—	1473 ± 922	—	62 ± 25	—	
Novotny, et al. (2012) [38] Am J Clin Nutr, United States	Control	—	132.2	—	—	90.5 ± 0.5	Values are least-square means ± pooled SEMs. Compared with control, the proportion of MEI as a whole decreased by ∼3% with the incorporation of 42 g almonds into the daily diet (*P* < 0.05) and by 5% with the incorporation of 84 g almonds into the daily diet (*P* < 0.05).
	Almonds (42 g/d)	—	217.7	—	—	87.5 ± 0.5	
	Almonds (84 g/d)	—	282.3	—	—	85.5 ± 0.5	
Baer et al. (2014) [42] J Nutr, United States	Placebo (0 g/d RM + 50 g/d maltodextrin)	107.0	129.9	236.9	95.6	92.0	Values are least-square means. The proportion of DEI and MEI was calculated from the mean of the results. Under conditions where energy intake was equalized across 3 groups, the addition of RM increased feces energy excretion (*P* < 0.05 compared with the control in each condition). Consequently, the proportion of DEI and MEI of RM25 and RM50 was also lower than that of the Control (RM0).
	RM (25 g/d RM + 25 g/d maltodextrin)	114.6	158.1	272.7	94.7	90.8	
	RM (50 g/d RM + 0 g/d maltodextrin)	110.3	176	286.3	94.0	90.3	
Baer, et al. (2016) [37] J Nutr, United States	Base diet	—	140 ± 8.9	—	—	90.4 ± 0.3	Values are least-square means ± pooled SEMs. The proportion of DEI decreased significantly during walnut consumption compared with the control phase (*P* < 0.0001).
	Walnuts		217 ± 8.9	—	—	87.8 ± 0.3	
Baer, et al. (2018) [39] Nutrients, United States	Control diet	118.2±5.0	129.6±8.1	248	94.9 ± 0.2	90.2	Values are means ± pooled SEMs. The proportion of MEI was calculated from the mean of the results. Although the literature referred to MEI as energy digestibility, the values approximated DEI, so it used as DEI. Energy digestibility of the diet decreased with the addition of cashews to the diet from 94.9% to 92.9% (*P* < 0.0001).
	Control diet + cashew diet	115.9±5.0	186.3±8.1	302	92.9 ± 0.2	88.4	
Basolo A, et al. (2020 [11]) Nat Med, United States	ALL	—	—	—	—	—	Values are expressed as mean (±SD). The absolute/proportion of MEI was calculated from the mean of intake and excretion from urine and feces. The proportion of DEI was significantly greater in overfeeding than underfeeding (94.2% ± 1.9% vs. 91.1% ± 3.7%, *P* < 0.0001), vancomycin led to a significant decrease in the proportion of DEI than placebo (91.6% ± 1.9% vs. 94.2% ± 2.2%, *P* = 0.0069).
	Underfeeding	33.9 ± 6.8	123.5 ± 32.4	157.4	91.1 ± 3.7	88.8	
	Overfeeding	47.7 ± 9.4	257.4 ± 91.2	305.1	94.2 ± 1.9	93.1	
	Male	—	—	—	—	—	
	Underfeeding	34.6 ± 7.1	134.1 ± 31.3	168.7	90.6 ± 4.5	88.4	
	Overfeeding	47.2 ± 9.2	281.1 ± 99.4	328.3	94.2 ± 2.1	93.2	
	Female	—	—	—	—	—	
	Underfeeding	32.9 ± 6.5	112.7 ± 31.7	145.6	91.9 ± 2.2	89.5	
	Overfeeding	48.5 ± 10.2	219.4 ± 76.5	267.9	94.2 ± 2.0	93.0	
	ALL	—	—	—	—	—	
	Vancomycin group	39.4 ± 8.6	260.1 ± 66.5	299.5	91.6 ± 1.9	90.3	
	Placebo	38.4 ± 7.8	176.8 ± 79.5	215.2	94.4 ± 2.5	93.1	
Bao, et al. (2022) [32] Front Endocrinol, China	Control	39.33	142.03±17.33	181.36	92.1	90.0	Values are expressed as mean (±SD). DEI and MEI were calculated from the mean of the results. TRE compared with control schedule is associated with a 22.7% increase in feces energy loss (Δ32.25 ± 9.33 kcal, *P* = 0.005) and a trend in increasing 14.5% urine energy loss (Δ6.67 ± 3.14 kcal, *P* = 0.058) without change energy expenditure.
	TRE	46.00	174.28±18.04	220.28	90.4	87.8	
Dawson, et al. (2024) [33] Cell Rep Med, United States	Early TRE	106 ± 28	229 ± 100	335	91.7 ± 2.3	87.8 ± 2.3	Values are expressed as mean (±SD). eTRE has no effect on intestinal energy compared with the control schedule (*P* = 0.95).
	Control	100 ± 22	228 ± 78	328	91.7 ± 1.5	88.0 ± 1.5	
Yoshimura, et al. (2024) [12] Obesity (Silver Spring), Japan	Overfeeding	102 (90, 114)	266 (241, 292)	368	92.6 (91.7, 96.5)	89.8 (88.8, 90.7)	Values are expressed as mean (95% Cl). The proportion of MEI significantly increased under overfeeding than under control (*P* < 0.05) and underfeeding (*P* < 0.05). However, the proportion of DEI did not differ between conditions.
	Control	85 (73, 97)	214 (187, 241)	299	91.5 (90.6, 92.4)	87.8 (86.8, 88.8)	
	Underfeeding	73 (61, 85)	159 (134, 185)	232	91.6 (90.7, 92.4)	87.7 (86.8, 88.7)	

Abbreviations: CI, confidence interval; DEI, digestible energy intake; HPN, home parenteral nutrition; LBRT, lower body resistance training; MEI, metabolizable energy intake; RM, resistant maltodextrin; RT6 (6 wk from intervention study); RT12 (12 wk from intervention study); TRE, time-restricted eating; WBRT, whole-body resistance training.

### Energy loads

Across 6 studies on overeating [[8], [9], [10], [11], [12],^22^], the proportions of DEI and MEI on mean levels were consistently higher in overeating conditions than in controls (Table 2). However, only 1 study reported a significant increase in the proportion of DEI under overeating condition compared with that under other conditions [11]. Jumpertz et al. [10] further observed that when comparing a 3400 kcal/d diet with a 2400 kcal/d diet, the proportion of DEI increased during overeating in lean individuals but showed no significant change in individuals with obesity. In contrast, an intervention on undereating found no dose-response relationship between the control diet and 2 levels of undereating [27].

### Dietary contents

Of 6 studies on dietary content, 4 examined the effects of high-fiber and low-fiber diets on the proportions of DEI and MEI [28,29,41,42], whereas 1 study compared low-glycemic and high-glycemic diets [30]. Across the 4 fiber studies [28,29,41,42], the proportions of DEI and/or MEI were consistently lower in the high-fiber diet than in the low-fiber diet (Table 2). In the 3 studies that tested statistical differences between conditions [29,41,42], high-fiber diets consistently showed lower proportion of DEI compared with low-fiber diets.

### Tree nuts

Across 4 intervention studies on tree nuts [31,[37], [38], [39]], the proportions of DEI and MEI decreased significantly during tree nuts consumption compared with those during controlled condition.

### Seasonal variation

One study evaluated seasonal changes (Spring, Summer, Fall, and Winter) in the proportions of DEI and MEI over a year, but did not present statistical comparisons between seasons [26].

### Exercise

In a study on the proportion of DEI and MEI with resistance training (lower body and whole-body resistance training), no significant group-by-time interactions were observed [40].

### Time-restricted eating

Two studies on TRE [32,33], reported that, compared with the control schedule, Bao et al. [32] indicated that TRE was associated with an increase in fecal EL and a trend toward an increase in urine EL, without changes in energy expenditure. However, Dawson et al. [33] found no difference in the proportions of DEI and MEI between TRE and control.

### Nonhealthy participants and aging

Among the 3 studies involving patients with SBS [34,36], or intestinal failure [35], 3 evaluated the effect of medication or supplementation on the proportion of DEI [11,34,36]. In patients with intestinal failure, a cross-sectional study [35] reported that those receiving home parenteral nutrition (HPN) had a significantly lower proportion of DEI than their counterparts (49% compared with 71%).

### The effects of medication or supplementation (vancomycin, cholylsarcosine, and colostrum)

**FIGURE 2:**
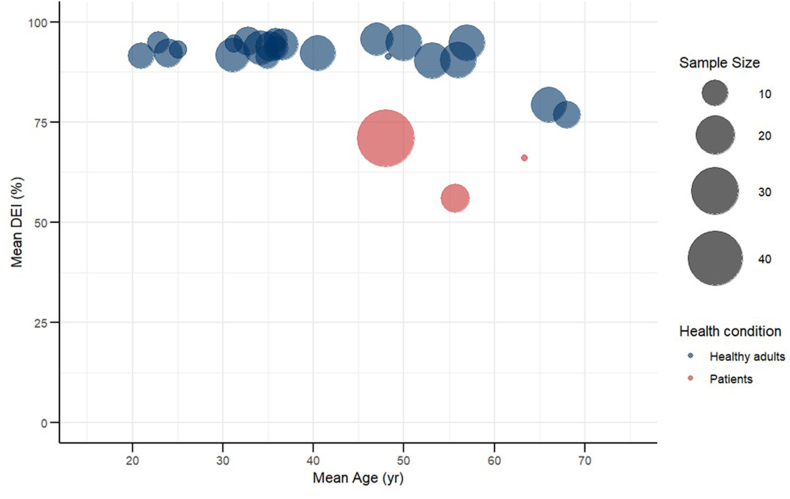
Bubble plot of mean age and mean digestible energy intake. Blue bubbles represent results from healthy adults, whereas red bubbles represent results from patients. The size of each bubble corresponds to the sample size. DEI, digestible energy intake.

The relationships among age, on an mean, DEI, and MEI are shown in Figure 2 and Supplementary Figure. In 1 study of healthy participants aged >60 y, stratified by sex, both the proportions of DEI (males 76.8%; females 79.3%) and MEI (males 74.1%; females 76.8%) were visually lower in younger participants than in their older counterparts [40]. Similarly, 3 studies on nonhealthy participants reported visually lower proportion of DEI (Heydorn et al. [34], 66%; Jeppesen et al. [35], 71%; Lund et al. [36], 62%) than in healthy individuals. Detailed data for each study’s participants are presented in the Supplementary Table 4.

The effect of supplementation of cholylsarcosine or colostrum on the proportion of DEI did not differ significantly between groups [34,36]. In addition, Basolo et al. [11], has shown that in healthy adults, the medication of vancomycin led to a significant reduction in the proportion of DEI compared with placebo (91.6% compared with 94.2%).

## Discussion

This systematic review evaluated 23 studies that used bomb calorimetry to assess digestible and ME by measuring GEI and energy excretion through feces and/or urine. The findings provide valuable insights into how different dietary interventions, including energy loads (overfeeding/underfeeding), tree nut intake, dietary fiber, and TRE, influence energy balance and absorption. Of the included studies, 21 were intervention trials, and 3 involved participants with health conditions such as SBS or intestinal failure.

### Impact of energy loads on the proportion of DEI and MEI

Studies on overeating consistently demonstrated that not all excess caloric intake is converted into weight gain. Across 6 studies, overeating was associated with higher absolute fecal EL, suggesting that adaptive mechanisms, such as enhanced thermogenesis and reduced nutrient absorption, help regulate energy balance [[8], [9], [10], [11], [12],^25^]. However, only 1 study reported a significant increase in the proportion of DEI between groups due to changes in dietary intake when overeating [11]. On mean, the proportions of DEI and MEI were higher in the overeating group than in the control group across all studies [[8], [9], [10], [11], [12],^25^]. When comparing overeating with underfeeding, Basolo et al. [11] reported a significantly higher DEI proportion in the underfeeding condition than in the overfeeding condition, although it remains unclear whether this reflects a reduction during underfeeding or an increase during overeating. Moreover, 1 study found no dose-response relationship between the control diet and different levels of undereating [27], and another failed to detect significant differences compared with control [43]. Considering the results of the previous studies on overeating, changes in the proportion of DEI due to energy load may be largely influenced by overeating.

In our previous study [43], fasting insulin concentrations were higher during overeating than in control and during undereating. Although insulin’s potent anabolic effects on protein, carbohydrate, and lipid metabolism [44,45] might partially explain the higher DEI during overeating, further research is needed. Nonetheless, the variability in methodologies across studies weakened the overall strength of the evidence, warranting further clinical trials to clarify the implications of these findings for clinical dietary practice

### Dietary contents

Two studies emphasized the role of dietary fiber in modulating MEI. Wisker et al. [28] and Baer et al. [29] demonstrated that high-fiber diets increased fecal EL by reducing the digestibility of fat and protein. The increase in fiber intake not only enhanced fecal bulk but also decreased the apparent digestibility of macronutrients, thereby lowering MEI. These findings underscore that dietary composition, particularly fiber content, can significantly alter the bioavailability of energy from foods. Corbin et al. [46] examined the effects of fiber intake on ME using calculated fecal EL and total energy intake in terms of chemical oxygen demand (COD). Because the present review restricted inclusion to studies using bomb calorimetry, COD-based approaches were excluded. Nevertheless, the COD method remains noteworthy, as it can be applied to estimate ME available to the host [47]. In addition, the proportion of DEI was consistently lower with high-fiber diets than with low-fiber diets, ranging from 84% to 96% in high-fiber conditions.

### Tree nut intake

Across 4 intervention studies on tree nuts [31,[37], [38], [39]], all reported a significant reduction in the proportion of DEI during tree nut consumption compared with control conditions, with differences ranging from 1.8% to 5.0%. Each study demonstrated a decrease in the percentage of digestible lipids [31,[37], [38], [39]], and 3 also reported reductions in the percentage of digestible proteins and carbohydrates compared with that in the control condition [31,38,39].

A systematic review by Nikodijevic et al. [48] further confirmed that the MEI of nuts, including almonds, cashews, hazelnuts, pistachios, walnuts, and peanuts, was consistently lower than values predicted by the Atwater factors. MEI varied according to tree nut type, physical form (flour > chopped > whole), extent of heat processing (butter > roasted > raw), and intake amount [48]. Collectively, these findings suggest that tree nut consumption reduces the bioavailability of major nutrients beyond carbohydrates and proteins. However, further studies are needed to clarify the mechanisms and quantify their clinical relevance.

### Time-restricted eating

Meal window reduction has gained attention in weight management due to its ease of implementation, and although TRE with and without dietary restriction [[49], [50], [51], [52]] affect body weight, the underlying mechanism remains unclear. In 2 crossover trials [32,33], evaluating the effect of TRE on energy absorption, the results were inconsistent. Dawson et al. [33], who established a 3-d adaptation period followed by a 9-d intervention in each condition, found no significant early TRE effect on intestinal nutrient absorption. However, Bao et al. [32] reported that a 1-d early TRE increased fecal EL, contributing to a negative energy balance. Both TRE studies employed in this study evaluated energy expenditure under controlled conditions using indirect calorimetry and found no difference between TRE and control. Because neither study detected changes in gut transit time, the discrepancy cannot be explained solely by changes in transit time. Both studies [32,33] involved healthy adults, but differences in participant characteristics [e.g., ethnicity (primarily White compared with Asian), BMI (24 ± 3 compared with 22 ± 2 kg/m^2^), energy intake] and intervention duration (9 d compared with 1 d) may also have contributed. Future studies should distinguish between acute and chronic effects of TRE to elucidate the mechanisms and time course of intestinal adaptation.

### Aging and disease

Visualized data suggested that older adults and nonhealthy participants with SBS showed reduced energy absorption. Of the 3 reports on SBS or intestinal failure, 2 studies examining the effect of bovine colostrum or cholylsarcosine found no significant differences in the proportion of DEI between intervention and control. In addition, a cross-sectional study reported that patients receiving HPN had a lower proportion of DEI than those not on HPN (49% compared with 71%, *P <* 0.01). Overall, the effects of aging and disease on energy absorption are insufficiently studied and should be examined further.

### Medication and supplementation

Basolo et al. [11] indicated that vancomycin use significantly decreased DEI compared with placebo (91.6% ± 1.9% compared with 94.2% ± 2.2%, Δ = 2.6%, *P =* 0.0069), with no significant differences in energy expenditure or substrate oxidation. Studies also suggest that drugs inhibiting lipid digestion in the intestinal tract (orlistat) or glucose reabsorption in the kidneys (SGLT2 inhibitors) may aid weight regulation; however, their effects on the proportion of DEI warrant further investigation in humans [53,54].

### Mechanisms associated with DEI and MEI regulation in the human intestinal tract

To better interpret our findings, it is important to consider potential physiological mechanisms underlying the observed differences in the proportions of DEI and MEI across dietary interventions, feeding patterns, and populations. For example, the reduced proportions of DEI and MEI with high-fiber diets and tree nut intake, inconsistent effects of TRE, and low values in older adults or diseased populations may be partly explained by alterations in intestinal nutrient absorption, microbial fermentation, and gut transit dynamics.

The gastrointestinal tract regulates energy balance primarily through nutrient absorption. In healthy adults, the majority of energy derived from dietary carbohydrates, lipids, and proteins are absorbed in the small intestine, although absorption efficiency varies considerably between individuals (83%–97%) [55]. This process primarily involves enzymatic digestion and transport mechanisms acting on carbohydrates, proteins, and lipids [28,29,33,46,[55], [56], [57], [58]]. Factors such as intestinal surface area and gastric emptying influence the efficiency of absorption in this region. Not all dietary components are fully digested in the small intestine. Complex carbohydrates and resistant starches that resist enzymatic breakdown reach the colon, where they undergo microbial fermentation. The resulting short-chain fatty acids (SCFAs)—mainly acetate, propionate, and butyrate—are absorbed by colonic epithelial cells and provide a modest energy contribution [46]. Beyond their caloric value, SCFAs exert broader metabolic effects, including roles in signaling and lipid regulation.

Fecal energy dynamics are governed not only by substrate availability but also by microbial activity. A substantial proportion of fermentation-derived energy may be allocated toward bacterial growth rather than host uptake. It has been suggested that up to half of fecal energy is embedded in microbial biomass [46,59,60]. Consequently, diets high in fermentable fiber might paradoxically reduce net energy absorption by increasing microbial proliferation, a mechanism that may contribute to weight regulation in some individuals.

Transit time through the colon further modulates these processes. Longer transit allows greater fermentation and potentially enhances energy harvest, whereas rapid transit limits microbial activity and increases fecal EL [46,61,62]. Experimental studies in germ-free mice also suggest that reduced SCFA availability may enhance nutrient absorption through compensatory hormonal responses such as increased glucagon-like peptide-1 secretion and slowed gut transit time [63]. However, these experimental results should be considered mechanistic context rather than direct evidence in humans.

### Strengths and limitations

To the best of our knowledge, this study is the first systematic review involving human participants to comprehensively examine DEI and MEI. Because of the wide range of intervention targets and the limited number of eligible articles, a meta-analysis was deemed inappropriate. Factors such as physical activity, stress, age, health status, and alcohol consumption may influence the amount of energy extracted from foods [2]. The use of bomb calorimetry in these studies provided a high degree of precision in measuring MEI. However, differences in methodologies, such as study setting (laboratory compared with free-living condition), sample collection duration, dietary control, and participant characteristics, may account for variability in the results. Moreover, this review focused solely on English literature, excluded participants aged <18 y, and included multiple studies with small sample sizes. These factors may limit the generalizability of our findings. Future studies would benefit from standardized protocols to improve comparability across studies.

### Future directions

It remains challenging to rigorously evaluate DEI using conventional fecal energy measurements with a bomb calorimeter, as these values also reflect energy contributions from intestinal bacteria and exfoliated epithelial cells. A complementary approach has been proposed, using polyethylene glycol as a nonabsorbable marker to evaluate energy absorption in the small intestine independently of the gut microbiota [46,64,65].

Of the 23 studies included in this review, 21 focused on young and middle-aged healthy adults (mean age <60 y). Energy absorption may vary across life stages and health conditions. One study on older adult residents (85 ± 7 y) in care facilities suggested the possibility of decreased energy absorption [66]. Elucidating DEI and MEI in older adults, particularly those with multimorbidity, will be essential for understanding mechanisms underlying frailty and sarcopenia in aging societies.

In conclusion, this review highlights the complexity of energy balance regulation and underscores the significant influence of dietary quantity and composition on the absolute/proportion of DEI and MEI. In addition, although the number of references is limited, the results suggest that the proportion of DEI and MEI declines with age or disease. Furthermore, research on the absolute/proportion of DEI and MEI, especially RCT, is warranted to clarify these associations.

## Author contributions

The authors’ responsibilities were as follows – EY, YN: designed research, analyzed data, and had primary responsibility for final content; and all authors: conducted research, wrote the paper, read and approved the final manuscript.

## Data availability

Data described in the manuscript, code book, and analytic code will be made available on request pending approval by the Ethics Committee.

## Declaration of generative AI and AI-assisted technologies in the writing process

During the preparation of this manuscript, the authors used DeepL to assist in editing grammar and improving language clarity.

## Funding

This study was supported by JSPS KAKENHI Grant Number 23K28033.

## Conflict of interest

The authors report no conflicts of interest.
