# Supplementary material for: Digestible and Metabolizable Energy Intake in Humans: a Systematic Review
Source: Adv Nutr. 2026 Feb 6;17(3):100597. doi: 10.1016/j.advnut.2026.100597 (PMC12955675; doi:10.1016/j.advnut.2026.100597)

**Supplementary Material 1**: Search strings used in databases.

Database: MEDLINE via PubMed

Search day:

#1 to #32 July 3, 2024

#33 to #34 September 18, 2025

| **#** | **Search items** | **Query** | **Results** |
| --- | --- | --- | --- |
| 1 | "bomb calorimetry" | "bomb calorimetry"[All Fields] | 196 |
| 2 | "bomb calorimeter" | "bomb calorimeter"[All Fields] | 96 |
| 3 | #1 or #2 |  | 288 |
| 4 | combust | "combust"[All Fields] OR "combusted"[All Fields] OR "combustibility"[All Fields] OR "combustible"[All Fields] OR "combustibles"[All Fields] OR "combusting"[All Fields] OR "combustion"[All Fields] OR "combustions"[All Fields] OR "combustive"[All Fields] OR "combusts"[All Fields] | 31153 |
| 5 | burn | "burns"[MeSH Terms] OR "burns"[All Fields] OR "burn"[All Fields] | 142459 |
| 6 | #4 or #5 |  | 172830 |
| 7 | feces | "faeces"[All Fields] OR "feces"[MeSH Terms] OR "feces"[All Fields] | 137759 |
| 8 | fecal | "faecally"[All Fields] OR "fecally"[All Fields] OR "fecals"[All Fields] OR "feces"[MeSH Terms] OR "feces"[All Fields] OR "faecal"[All Fields] OR "fecal"[All Fields] | 200354 |
| 9 | dejection | "dejected"[All Fields] OR "dejection"[All Fields] OR "dejections"[All Fields] | 454 |
| 10 | flux | "flux"[All Fields] | 103731 |
| 11 | #7 or #8 or #9 or #10 |  | 309996 |
| 12 | urine | "urine"[MeSH Subheading] OR "urine"[All Fields] OR "urine"[MeSH Terms] OR "urines"[All Fields] | 406207 |
| 13 | #11 and #12 |  | 20294 |
| 14 | #6 and #13 |  | 110 |
| 15 | absorption | "absorptance"[All Fields] OR "absorptances"[All Fields] OR "absorption"[MeSH Terms] OR "absorption"[All Fields] OR "absorptions"[All Fields] OR "absorptive"[All Fields] OR "absorptivities"[All Fields] OR "absorptivity"[All Fields] | 401822 |
| 16 | malabsorption | "malabsorption"[All Fields] OR "malabsorptions"[All Fields] OR "malabsorptive"[All Fields] | 18807 |
| 17 | #15 or #16 |  | 415736 |
| 18 | energy | "energie"[All Fields] OR "energies"[All Fields] OR "energy"[All Fields] | 1145322 |
| 19 | #17 and #18 |  | 57553 |
| 20 | #13 and #19 |  | 128 |
| 21 | "energy loss" | "energy loss"[All Fields] | 7773 |
| 22 | "caloric loss" | "caloric loss"[All Fields] | 37 |
| 23 | #21 or #22 |  | 7810 |
| 24 | "diet intake" | "diet intake"[All Fields] | 1144 |
| 25 | "dietary intake" | "dietary intake"[All Fields] | 33069 |
| 26 | "food intake" | "food intake"[All Fields] | 54390 |
| 27 | "energy intake" | "energy intake"[All Fields] | 59304 |
| 28 | "caloric intake" | "caloric intake"[All Fields] | 8248 |
| 29 | #24 or #25 or #26 or #27 or #28 |  | 136762 |
| 30 | #23 and #29 |  | 113 |
| 31 | #3 or #14 or #20 or #30 |  | 623 |
| 32 | #31 Filters: from 1973- |  | 599 |
| 33 | "apparent metabolizable energy"  Filters: from 1973-2024/7 | "apparent metabolizable energy"[All Fields] AND 1973/01/01:2024/07/31[Date - Publication] | 274 |
| 34 | #32 and #33 |  | **873** |

Database: Cochrane

Search day:

#1 to #15 July 3, 2024

#16 to #17 September 18, 2025

| **#** | **Query *** | **Results** |
| --- | --- | --- |
| 1 | ("bomb calorimetry") OR ("bomb calorimeter") | 28 |
| 2 | (combust) OR (burn) | 5802 |
| 3 | (feces) OR (fecal) OR (dejection) OR (flux) | 21645 |
| 4 | (urine) | 53997 |
| 5 | #3 and #4 | 2271 |
| 6 | #2 and #5 | 13 |
| 7 | (absorption) OR (malabsorption) | 22876 |
| 8 | (energy) | 46694 |
| 9 | #7 AND #8 | 1153 |
| 10 | #5 AND #9 | 43 |
| 11 | ("energy loss") OR ("caloric loss") | 85 |
| 12 | ("diet intake") OR ("dietary intake") OR ("food intake") OR ("energy intake") OR ("caloric intake") | 28398 |
| 13 | #11 AND #12 | 16 |
| 14 | #1 OR #6 OR #10 OR #13 | 88 |
| 15 | #14 Filters: from 1973- | 72 |
| 16 | ("apparent metabolizable energy")  Filters: from 1973-2024 | 5 |
| 17 | #15 and #16 | **77** |

*All search items were set to 'All text' during the search.

Database: CINAHL

Search day:

S1 to S15 July 3, 2024

S16 to S17 September 18, 2025

| **S** | **Query*** | **Results** |
| --- | --- | --- |
| 1 | ("bomb calorimetry") OR ("bomb calorimeter") | 33 |
| 2 | (combust) OR (burn) | 43447 |
| 3 | (feces) OR (fecal) OR (dejection) OR (flux) | 26466 |
| 4 | (urine) | 43879 |
| 5 | S3 and S4 | 1477 |
| 6 | S2 and S5 | 26 |
| 7 | (absorption) OR (malabsorption) | 19532 |
| 8 | (energy) | 102751 |
| 9 | S7 AND S8 | 1658 |
| 10 | S5 AND S9 | 24 |
| 11 | ("energy loss") OR ("caloric loss") | 261 |
| 12 | ("diet intake") OR ("dietary intake") OR ("food intake") OR ("energy intake") OR ("caloric intake") | 57952 |
| 13 | S11 AND S12 | 11 |
| 14 | S1 OR S6 OR S10 OR S13 | 90 |
| 15 | S14 Filters: from 1973- | 90 |
| 16 | "apparent metabolizable energy" Filter: 1973/1/1-2024/7/3 | 52 |
| 17 | S16 AND S17 | **142** |

*All search items were set to 'TX' during the search.

Database: Scopus

Search day:

#1 to #16 conducted on July 8, 2024

#17 to #18 conducted on September 18, 2025

| **#** | **Query** | **Results** |
| --- | --- | --- |
| 1 | ( TITLE-ABS ( "bomb calorimetry" ) OR TITLE-ABS ( "bomb calorimeter" ) ) | 1883 |
| 2 | ( TITLE-ABS ( combust ) OR TITLE-ABS ( burn ) ) | 138421 |
| 3 | ( TITLE-ABS ( feces ) OR TITLE-ABS ( fecal ) OR TITLE-ABS ( dejection ) OR TITLE-ABS ( flux ) ) | 979016 |
| 4 | TITLE-ABS ( urine ) | 342732 |
| 5 | #3 AND #4 | 19729 |
| 6 | #2 AND #5 | 31 |
| 7 | ( TITLE-ABS ( absorption ) OR TITLE-ABS ( malabsorption ) ) | 1306042 |
| 8 | TITLE-ABS ( energy ) | 5421964 |
| 9 | #7 AND #8 | 263503 |
| 10 | #5 AND #9 | 100 |
| 11 | ( TITLE-ABS ( "energy loss" ) OR TITLE-ABS ( "caloric loss" ) ) | 76011 |
| 12 | ( TITLE-ABS ( "diet intake" ) OR TITLE-ABS ( "dietary intake" ) OR TITLE-ABS ( "food intake" ) OR TITLE-ABS ( "energy intake" ) OR TITLE-ABS ( "caloric intake" ) ) | 145704 |
| 13 | #11 AND #12 | 246 |
| 14 | #1 OR #6 OR #10 OR #13 | 2247 |
| 15 | #31 Filters: Article | 1980 |
| 16 | #31 Filters: from 1973-2024 | 1747 |
| 17 | TITLE-ABS ( "apparent metabolizable energy" ) AND PUBYEAR > 1973 AND PUBYEAR < 2024 | 783 |
| 18 | #16 AND #17 | **2530** |

**Supplemental Table 1. Critical appraisal results of randomized controlled trials**

Risk of bias in randomized controlled trials based on JBI Critical Appraisal Checklist for randomized controlled trials. Y, yes; N, no; NA, not applicable; U, unclear. Q1. Was true randomization used for assignment of participants to treatment groups? Q2. Was allocation to treatment groups concealed? Q3. Were treatment groups similar at the baseline? Q4. Were participants blind to treatment assignment? Q5. Were those delivering treatment blind to treatment assignment? Q6. Were outcomes assessors blind to treatment assignment? Q7. Were treatment groups treated identically other than the intervention of interest? Q8. Was follow up complete and if not, were differences between groups in terms of their follow up adequately described and analyzed? Q9. Were participants analyzed in the groups to which they were randomized? Q10. Were outcomes measured in the same way for treatment groups? Q11. Were outcomes measured in a reliable way? Q12. Was appropriate statistical analysis used? Q13. Was the trial design appropriate, and any deviations from the standard RCT design (individual randomization, parallel groups) accounted for in the conduct and analysis of the trial?

| **Author** | **Year** | **Q1** | **Q2** | **Q3** | **Q4** | **Q5** | **Q6** | **Q7** | **Q8** | **Q9** | **Q10** | **Q11** | **Q12** | **Q13** |
| --- | --- | --- | --- | --- | --- | --- | --- | --- | --- | --- | --- | --- | --- | --- |
| Wisker, et al. | 1988 | No | Unclear | Yes | No | No | Unclear | Yes | Yes | Yes | Yes | No | Yes | Yes |
| Baer, et al. | 1997 | Unclear | Unclear | Unclear | No | No | Unclear | Yes | Yes | Yes | Yes | No | Yes | Yes |
| Heydorn, et al. | 1999 | Unclear | Unclear | Unclear | Unclear | Unclear | Unclear | No | Yes | No | Yes | No | No | No |
| Campbell, et al. | 2002 | Unclear | Unclear | Yes | No | No | Unclear | Yes | Yes | Yes | Yes | No | Yes | Yes |
| Clapp, et al. | 2007 | Unclear | Unclear | Yes | No | No | Yes | Yes | No | No | Yes | Yes | Yes | Yes |
| Zou, et al. | 2007 | Unclear | Unclear | Unclear | No | No | Unclear | Yes | Yes | Yes | Yes | Yes | Yes | Yes |
| Jumpertz, et al. | 2011 | Unclear | Unclear | Yes | No | No | Unclear | Yes | Yes | Yes | Yes | Yes | Yes | Yes |
| Baer, et al. | 2012 | Unclear | Yes | Yes | No | No | Unclear | Yes | Yes | No | Yes | Yes | Yes | No |
| Lund, et al. | 2012 | Unclear | Unclear | Unclear | Yes | Yes | Unclear | Yes | Yes | No | Yes | No | Yes | Yes |
| Novotny, et al. | 2012 | No | Unclear | Unclear | No | No | Unclear | Yes | Yes | Yes | Yes | Yes | Yes | Yes |
| Baer, et al. | 2014 | Unclear | Unclear | Unclear | Yes | Yes | Unclear | Yes | Yes | No | Yes | Yes | Yes | Yes |
| Baer, et al. | 2016 | Yes | Unclear | Unclear | No | No | Unclear | Yes | Yes | Yes | Yes | Yes | Yes | Yes |
| Baer, et al. | 2018 | Unclear | Unclear | Unclear | No | No | Unclear | Yes | Yes | Yes | Yes | Yes | Yes | Yes |
| Basolo, et al. cross-over trial | 2020 | Yes | Yes | Unclear | No | No | Unclear | Yes | Yes | Yes | Yes | Yes | Yes | Yes |
| Basolo, et al. randomized control trial | 2020 | Yes | Yes | Yes | Yes | Yes | Unclear | Yes | Yes | No | Yes | Yes | Yes | Yes |
| Bao, et al. | 2022 | Yes | Unclear | Yes | No | No | Unclear | Yes | Yes | Yes | Yes | Yes | Yes | Yes |
| Dawson, et al. | 2024 | Yes | Unclear | Yes | No | No | No | Yes | Yes | Yes | Yes | Yes | Yes | Yes |
| Yoshimura, et al. | 2024 | Yes | Unclear | Unclear | No | No | Unclear | Yes | Yes | No | Yes | Yes | Yes | Yes |

**Supplemental Table 2. Critical appraisal results of** **analytical cross-sectional studies**

Risk of bias in cross-sectional studies based on JBI Critical Appraisal Checklist for analytical cross-sectional studies. Y, yes; N, no; NA, not applicable; U, unclear. Q1. Were the criteria for inclusion in the sample clearly defined? Q2. Were the study subjects and the setting described in detail? Q3. Was the exposure measured in a valid and reliable way? Q4. Were objective, standard criteria used for measurement of the condition? Q5. Were confounding factors identified? Q6. Were strategies to deal with confounding factors stated Q7. Were the outcomes measured in a valid and reliable way? Q8. Was appropriate statistical analysis used?

| **Author** | **Year** | **Q1** | **Q2** | **Q3** | **Q4** | **Q5** | **Q6** | **Q7** | **Q8** |
| --- | --- | --- | --- | --- | --- | --- | --- | --- | --- |
| Miles, et al. | 1984 | Unclear | Unclear | Yes | Yes | No | No | Yes | No |
| Jeppesen, et al. | 2000 | Yes | Yes | Yes | Yes | No | No | Yes | Yes |

**Supplemental Table 3. Critical appraisal results of quasi-experimental studies**

Risk of bias in one-arm intervention and experimental intervention studies based on JBI Critical Appraisal Checklist for quasi-experimental studies Y, yes; N, no; NA, not applicable; U, unclear. Q1. Is it clear in the study what is the “cause” and what is the “effect” (ie, there is no confusion about which variable comes first)? Q2. Was there a control group? Q3. Were participants included in any comparisons similar? Q4. Were the participants included in any comparisons receiving similar treatment/care, other than the exposure or intervention of interest? Q5. Were there multiple measurements of the outcome, both pre and post the intervention/exposure? Q6. Were the outcomes of participants included in any comparisons measured in the same way? Q7. Were outcomes measured in a reliable way? Q8. Was follow-up complete and if not, were differences between groups in terms of their follow-up adequately described and analyzed? Q9. Was appropriate statistical analysis used?

| **Author** | **Year** | **Q1** | **Q2** | **Q3** | **Q4** | **Q5** | **Q6** | **Q7** | **Q8** | **Q9** |
| --- | --- | --- | --- | --- | --- | --- | --- | --- | --- | --- |
| Webb, et al. | 1983 | Yes | No | No | Yes | No | Yes | No | Yes | Yes |
| Dallosso, et al. | 1984 | Yes | Yes | Yes | Yes | No | Yes | No | Yes | Yes |
| Webb, et al. | 1985 | Yes | No | Yes | No | No | Yes | No | Yes | No |
| Miles, et al. | 1986 | Yes | No | Yes | Yes | No | Yes | No | Yes | Yes |

**Supplemental Table 4. Critical appraisal results of quasi-experimental studies**

| **No** | **References** | **Disease status** | **Study condition** | **N** | **Mean age** | **DEI** | **MEI** |
| --- | --- | --- | --- | --- | --- | --- | --- |
| #1 | Webb (1983) | Healthy adults | Average diet control | 4 | 48.3 | 91.3 | 88.2 |
| #2 | Dallosso (1984) | Healthy adults | Control | 8 | 22.9 | 94.8 | 91.6 |
| #3 | Miles (1984)_A | Healthy adults | Male_all seasons | 13 | 35.3 | 93.9 | 89.5 |
| #4 | Miles (1984)_B | Healthy adults | Women_all seasons | 16 | 34.2 | 93.5 | 89.1 |
| #5 | Webb (1985) | Healthy adults | Control | 17 | 40.5 | 92.1 | 88.4 |
| #6 | Miles (1986) | Healthy adults | Control | 9 | 34.9 | 91.3 | 86.1 |
| #7 | Wisker (1988) | Healthy adults | Low fiber diet | 6 | 25 | 93.2 | 88 |
| #8 | Baer (1997) | Healthy adults | Medium fat, medium fiber | 6 | 31.2 | 94.6 | 90.3 |
| #9 | Heydorn (1999) | Patients | Cholylsarcosine (0g/day) | 4 | 63.3 | 66 | - |
| #10 | Jeppesen (2000) | Patients | Non-HPN | 44 | 48 | 71 | - |
| #11 | Campbell (2002)_A | Healthy adults | Men_baseline | 11 | 68 | 76.8 | 74.1 |
| #12 | Campbell (2002)_B | Healthy adults | Women_baseline | 17 | 66 | 79.3 | 76.8 |
| #13 | Clapp (2007) | Healthy adults | Low-glycemic index | 7 | 35 | - | 87.4 |
| #14 | Zou (2007) | Healthy adults | Refined diet | 9 | 35.9 | 93.5 | 90.3 |
| #15 | Jumpertz (2011)_A | Healthy adults | Lean_2400kcal/day | 12 | 32.8 | 95.1 | 91.9 |
| #16 | Jumpertz (2011)_B | Healthy adults | Obese_3400kcal/day | 9 | 35.8 | 95.4 | 92.5 |
| #17 | Baer (2012) | Healthy adults | Control | 18 | 50 | 94.9 | 89.5 |
| #18 | Lund (2012) | Patients | Baseline 1 (Water) | 12 | 55.7 | 56 | - |
| #19 | Novontny (2012) | Healthy adults | Control | 18 | 56 | 90.5 | - |
| #20 | Baer (2014) | Healthy adults | Placebo (0 g/d RM + 50 g/d maltodextrin) | 15 | 47 | 95.6 | 92 |
| #21 | Baer (2016) | Healthy adults | Base diet | 18 | 53.1 | 90.4 | - |
| #22 | Baer (2018) | Healthy adults | Control diet | 18 | 56.9 | 94.9 | 90.2 |
| #23 | Basolo (2020) | Healthy adults | Placebo | 14 | 36.6 | 94.4 | 93.1 |
| #24 | Bao (2022) | Healthy adults | Control | 12 | 24 | 92.1 | 90 |
| #25 | Dawson (2024) | Healthy adults | Control | 16 | 31.1 | 91.7 | 88 |
| #26 | Yoshimura (2024) | Healthy adults | Control | 10 | 21 | 91.5 | 87.8 |

**DEI**, digestible energy intake; **MEI**, metabolizable energy intake.

**Supplemental Figure.** Bubble plot of mean age and mean metabolizable energy intake. MEI, metabolizable energy intake.


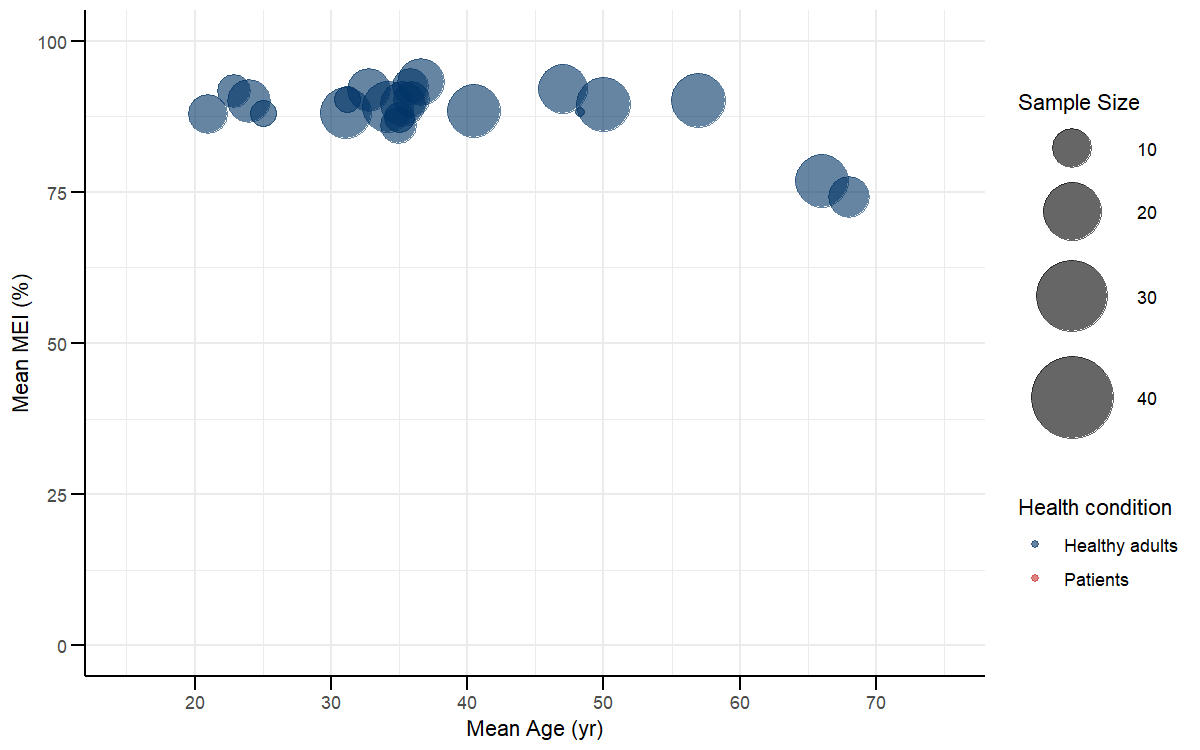

Supplement: multimedia component 1 [file mmc1.docx]
